# Effects of Tributyrin Supplementation in High-Soybean-Meal Diet on Growth Performance, Hemolymph and Hepatopancreas Immune and Antioxidant Parameters, and Intestinal Morphology of Juvenile Pacific White Shrimp (*Litopenaeus vannamei*)

**DOI:** 10.3390/ani16101496

**Published:** 2026-05-13

**Authors:** Sami Ullah, Minjie Zhao, Xiaomin Deng, Wu Jingci, Dong Yafei, Lin Danhua, Ying Liu, Fengqin Feng

**Affiliations:** 1 College of Biosystems Engineering and Food Science, Zhejiang University, Hangzhou 310058, China; samiwazir04@yahoo.com (S.U.); minjiezhao@zju.edu.cn (M.Z.); 2 Beijing Muyom Biotechnology Co., Ltd., Daxing District, Beijing 102627, China; bjmuyom@163.com; 3 Zhejiang University Zhongyuan Institute, Zhengzhou 450001, China; 17737416812@163.com (W.J.); 17796669538@139.com (D.Y.); xubei880828@163.com (L.D.)

**Keywords:** *Litopenaeus vannamei*, growth performance, tributyrin, antioxidant parameters, textural properties, Intestinal development

## Abstract

The effects of dietary tributyrin (TB) supplementation in juvenile *Litopenaeus vannamei* fed a high-soybean-meal diet were evaluated in an 8-week growth trial. The basal diet contained 24% fishmeal, while the experimental diet was supplemented with TB at 2.24 g kg^−1^. Tanks were randomly assigned each dietary treatment in triplicate, with each tank consisting of 50 shrimp with 1.66 ± 0.24 g as an initial weight. Shrimp fed with the control diet exhibited the lowest growth, whereas supplementation with TB improved growth performance. For ADCs (apparent digestibility coefficients), non-significant variances were detected in dry matter, crude lipid, and crude protein; however, ash was highly significant in the control (0.00) group. Moreover, gross energy was highly significant in the TB-supplemented group. Whole-body and dorsal muscle proximate composition showed no substantial differences (*p* > 0.05), except that ash was highly significant in the whole body for the control diet, while lipids were highly significant in dorsal muscles for the TB diet. The biochemical, antioxidant and immune parameters of the hepatopancreas and hemolymph showed non-significant changes in both groups, except total protein (TP) and triglycerides (TGs) were highly significant in the TB-supplemented group in both the hemolymph and hepatopancreas. Conversely, malondialdehyde (MDA) was highly significant in the control group in the hemolymph. Non-significant changes were detected among treatment groups for most of the textural properties of the shrimp; however, hardness was highly significant in the control group. The TB group showed a highly significantly larger intestinal villus height (*p* < 0.05). Compared with shrimp fed a control diet, those receiving the tributyrin-supplemented diet showed improved gut morphology and structure, as well as enhanced histological characteristics of the hepatopancreas. Overall, TB supplementation significantly enhanced the specific growth rate, weight gain rate, final body weight, and intestinal morphology. Although some indicators were not affected, these results indicate that TB has potential as a valuable dietary supplement in aquaculture.

## Simple Summary

This study investigated the effects of tributyrin on growth performance; nutrient digestibility; the proximate composition of the whole body and dorsal muscle; immune and antioxidant functions in the hemolymph and hepatopancreas; and intestinal morphology in the Pacific white shrimp *Litopenaeus vannamei*. The inclusion of tributyrin (TB) in the diet notably enhanced epithelial fold length and the number of goblet cells in the anterior intestine. Additionally, this study also assessed the textural properties of shrimp. The findings suggest that a 2.24 g dosage of TB is highly beneficial. These results have the potential to improve the health of aquatic species, reduce aquaculture costs, and lessen environmental impacts. Furthermore, this study indicates that tributyrin is a good dietary supplement for shrimp.

## 1. Introduction

The steady expansion of aquaculture production over recent decades, together with its projected growth in the coming years, presents numerous challenges for this sector. Among these challenges, industry sustainability remains a main concern, particularly due to continued dependence on aquatic stocks for the production of fish oil and fishmeal, which are key ingredients in aquafeeds [1]. This issue is particularly critical for aquaculture species; therefore, the identification and evaluation of alternative feed ingredients have been major focuses of aquaculture nutrition research over the past several decades [2,3]. However, regarding the species-wide diversity currently produced in aquatic farming [1], it is not only the expansion of nutritional requirement range and eating strategies that must be considered but also increases in the probability of species-specific physiological responses to a given experimental ingredient.

Plant diets have been extensively evaluated and optimized for a range of species of shrimp, and substantial progress has been made in recent years. Nevertheless, several limitations associated with their use remain. Constraints on the additional levels of plant-derived ingredients are commonly linked to essential amino acid deficiencies, particularly those in lysine and methionine [4], as well as the presence of antinutritional factors, including saponins, tannins, phytate, and lectins [5]. In addition, plant-based formulations are typically deficient in long-chain n-3 polyunsaturated fatty acids, namely docosahexaenoic acid (DHA) and eicosapentaenoic acid (EPA), and may result in imbalanced n-6/n-3 fatty acid [6,7]. Collectively, these nutritional deficiencies can impair growth performance and compromise the health status of shrimp. Moreover, plant-based diets have been associated with a pro-inflammatory intestinal environment, promoting gut inflammation, structural damage, and the development of enteritis [8,9,10,11,12,13]. Short-chain fatty acids, mostly butyric acid, and its derivatives like sodium butyrate, are also important in promoting intestinal health by improving epithelial cell proliferation and improving intestinal function [14]. Butyrate has been shown to improve immune function in fish fed plant diets [15,16,17].

Tributyrin (TB) is a triglyceride consisting of a glycerol backbone esterified with three molecules of butyric acid. Following enzymatic hydrolysis in the small intestine, one molecule of tributyrin releases three molecules of butyrate, which are readily absorbed by the intestinal epithelium [18,19]. Tributyrin supplementation has been shown to have beneficial effects on intestinal health and growth in pigs and rats [4,5,6,20,21,22]. It has been extensively investigated and is widely utilized as a functional feed additive in production animals, particularly in livestock species. In contrast, its incorporation into aquafeeds is a relatively recent development and has been less extensively investigated; nevertheless, research interest in this compound has steadily increased. Compared with butyrate, tributyrin (TB) exhibits stronger and more direct cellular effects, as well as improved pharmacokinetic properties [23]. In animals, roughly 90% of absorbed butyric acid is converted into ketone bodies [24]. Lane et al. [25] reported that the ketogenic capacity of the ruminal epithelium increases with age and that the expression of genes involved in ketogenesis occurs independently of ruminal butyric acid concentrations. Considering the critical role of microbial fermentation, promoting optimal microbial growth and metabolite production is essential. Due to their central role in fermentation, it is essential to optimize microbial growth and the production of metabolites. Butyrate has been shown to improve the immune response in fish consuming plant-based diets [15,16,17], thereby contributing to the mitigation or reversal of adverse effects associated with the inclusion of plant-derived ingredients in aquafeeds [14]. We hypothesized that tributyrin (TB) supplementation would beneficially modulate the gut microbiota. To investigate this, in vivo and in vitro experiments were performed to evaluate its effects on the protein synthesis of microbiota, nutrient degradability, and fermentation characteristics in adult Small-Tail ewes.

However, the impact of tributyrin supplementation on Pacific white shrimp has not been investigated to date. Therefore, the current study was designed to assess dietary tributyrin supplementation effects on growth performance; apparent nutrient digestibility; biochemical, antioxidant, and immune responses; muscle textural properties; intestinal mucosal morphology; and hepatopancreas histology in Pacific white shrimp (*Litopenaeus vannamei*).

## 2. Materials and Analysis Methods

### 2.1. Dietary Preparation and Feed Formulation

Tributyrin (TB), containing 62.5% active ingredient, was supplied by the South China University of Technology (Guangzhou, Guangdong, China) as a white powder. As a nutritional requirement of Pacific white shrimp, isonitrogenous and isoenergetic (40.21% crude protein and 17.89 kJ/g gross energy) diets were prepared. These included a control diet (TB 0.00) and an experimental diet supplemented with 2.24 g of TB. Soybean protein concentrate, soybean meal, fishmeal, and shrimp meal are the main protein sources in the diets. α-Starch was incorporated to maintain the isonitrogenous and isoenergetic properties of the diets. The formulation and proximate composition of experimental diets are shown in Table 1.

Table 2 presents the amino acid composition of the experimental diets. Before mixing and weighing, to ensure homogeneity, these ingredients were ground through a 178 μm mesh. The thoroughly blended mixture was then pelleted to a diameter of 1.2 mm using a (Modle HKJ-218, HUARUI, Wuxi, China) feed pelletizer. The resulting pellets were steamed for 10 min and subsequently air-dried under controlled conditions for 72 h. All diets were stored at minus 20 °C until use.

### 2.2. Experimental Design

Healthy Pacific white shrimp were obtained from the Mariculture Research Institute, Zhejiang Province, China. This feeding trial was conducted on Xixuan Island, Zhoushan, China.

Before the experimental trial, Pacific white shrimp were acclimatized to the rearing conditions for four weeks and fed a commercial diet. After a 24 h fasting period, 300 healthy *Litopenaeus vannamei* with an initial mean body weight of 1.66 ± 0.24 g were randomly assigned to six fiberglass tanks (500 L capacity per tank; 50 shrimp per tank). The control and tributyrin dietary treatments were each assigned to three replicate tanks.

During the 8-week feeding trial, *Litopenaeus vannamei* were cultured in a well-designed flow-through system supplied continuously with filtered seawater. Shrimp were fed four times daily at 06:00, 10:00, 14:00, and 18:00 under natural photoperiod conditions. Uneaten feed and feces were removed daily to maintain water quality. The following water quality parameters were monitored and maintained within optimal ranges: temperature 26–28 °C, total ammonia nitrogen 0.3–0.5 mg L^−1^, dissolved oxygen ≥6.5 mg L^−1^, pH 8.0–8.3, and 27.2–30.0 g/L salinity. These conditions were maintained to support the growth and survival of *Litopenaeus vannamei*.

### 2.3. Evaluation of Digestibility

Starting from the sixth week, shrimp feces were collected from each tank every morning at 6:00 a.m., prior to the next feeding. Fecal samples were collected following the methods described by Zhou et al. [26] and stored at −20 °C for subsequent analysis.

### 2.4. Collection of Samples

At the end of the 8-week feeding trial, all surviving shrimp were counted and weighed after a 24 h fasting period. From each tank, 15 shrimp were randomly selected for the measurement of body length, total hepatopancreas weight, and body weight. Furthermore, 10 shrimp were randomly collected from each tank for proximate composition analysis.

The remaining shrimp were euthanized, and dorsal muscle, hepatopancreas, and gut samples were collected. Hemolymph was withdrawn from the pericardial sinus located at the base of the first abdominal segment using a 1 mL disposable syringe. Hemolymph samples were collected and immediately mixed (1:1, *v*/*v*) with a precooled anticoagulant solution containing 0.16 M Na_2_HPO_4_, 0.34 M NaCl, 0.02 M EDTA, and 0.04 M NaH_2_PO_4_ (pH 7.4). Plasma for biochemical analyses was obtained by centrifugation at 3500× *g* for 10 min at 4 °C and was immediately snap-frozen in liquid nitrogen. Hepatopancreas samples were also rapidly frozen in liquid nitrogen for biochemical analyses. All samples stored in liquid nitrogen were subsequently transferred to −80 °C until further use. For histological examination, hepatopancreas tissues were fixed in 10% (*v*/*v*) neutral buffered formalin for 24 h and then preserved in 70% ethanol until processing.

### 2.5. Sample Analytical Methods

The moisture, ash, crude lipid, and crude protein contents of the diets, as well as those of the dorsal muscle and whole shrimp, were determined according to the methods of the Association of Official Analytical Chemists (AOAC) [27]. The amino acid composition of both the diets and whole shrimp was analyzed using an (Hitachi LA8080, Tokyo, Japan) automatic amino acid analyzer following acid hydrolysis with hydrochloric acid, as previously described [28].

### 2.6. Biochemical Assay Procedures

The hepatopancreas was homogenized in phosphate-buffered saline (PBS) at a 1:9 ratio, and then for 10 min, it was centrifuged at 4000× *g* at 4 °C. The resulting supernatants were used to measure various biochemical parameters using appropriate assay kits. Complement protein 3 (C3) was quantified using a specific kit from the same supplier. The levels of triglycerides (TGs) (A110-1-1), aspartate aminotransferase (AST) (C010-2-1), total cholesterol (T-CHO) (A111-1-1), and lysozyme (LZM) (A050-1-1) were measured using kits. Superoxide dismutase (SOD) (A001-3), malondialdehyde (MDA) (A003-1), and total antioxidant capacity (T-AOC) (A015-1) in the hepatopancreas were assessed using the kits (Jiancheng Bioengineering Institute, Nanjing, China). Total protein (TP) content was determined with the BCA Protein Assay Kit (A045-3, Jiancheng Bioengineering Institute, Nanjing, China).

### 2.7. Hematoxylin and Eosin (H&E) Staining Protocol for Hepatopancreas Tissue

#### Hematoxylin and Eosin (H&E) Staining Samples

The hepatopancreas samples were initially dehydrated through a series of graded ethanol, followed by xylene treatment, then embedded with paraffin wax, as described by F. Gao et al. (2019). The paraffin-embedded tissue slabs were sectioned into 5 μm thick slices, stained with hematoxylin and eosin (H&E), and examined using an (Nikon Ni-U, Tokyo, Japan) optical microscope.

### 2.8. Formulas and Statistical Analysis

The following equations were used to evaluate growth performance and feed efficiency:

IBW = initial mean body weight.

FBW = final mean body weight.

Weight gain (WG, %) = 100 × (final body weight − initial bodyweight)/initial body weight. 3 SGR (Specific growth rate) = 100 × (ln final body weight − ln initial body weight)/days.

MFI (Mean feed intake) = air drying diet fed in g/(shrimp × day).

FCR (Feed conversion ratio) = dry diet fed in g/wet weight gain in g.

CF (Condition factor) = 100 × (final body weight, g)/(body length, cm^3^).

HSI (Hepatosomatic index) = 100 × (hepatopancreas weight/body weight).

VSI (Viscerosomatic index) = viscera weight/body weight × 100.

SR (Survival rate) = 100 × (final shrimp number/initial shrimp number).

The results were presented as means ± standard deviation (SD). Statistical analysis was carried out using SPSS Statistics 27.00 software (IBM Analytics, Chicago, IL, USA). Comparisons between groups were analyzed using a one-way analysis of variance (ANOVA), followed by the multiple comparison test of Dunnett. *p* < 0.05 was considered to be statistically significant.

## 3. Results

### 3.1. Growth Performance and Body Condition

Table 3 shows growth performance indicators. The final body weight, weight gain, and specific growth rate were significantly greater in the TB group. In contrast, the hepatosomatic and viscerosomatic indices were highly significant in the control group. There were no significant differences observed between the groups in initial body weight, condition factor, mean feed intake, feed conversion ratio, or survival rate. These findings suggest that TB had a notable impact on shrimp growth performance.

### 3.2. Apparent Digestibility of Nutrients

As shown in Table 4, the apparent digestibility coefficients of crude lipid, crude protein, dry matter, ash, and gross energy were evaluated. Compared to the TB group, ash content was significantly higher in the control group. There were no significant differences detected among treatments in the apparent digestibility coefficients (ADCs) of crude protein, crude lipid, and gross energy.

### 3.3. Analysis of Whole-Body and Muscle Proximate Composition

Table 5 shows the dorsal muscle and whole-body proximate composition. Non-significant differences were detected among groups in whole-body lipid, protein, moisture, and phosphorus content (*p* > 0.05). However, ash content was significantly higher in the control group compared to treated groups (*p* < 0.05). In the dorsal muscle, non-significant differences were found among groups in protein, moisture, ash, and phosphorus content (*p* > 0.05). In contrast, the crude lipid content in the treated group was highly significant compared to the control group (*p* < 0.05).

### 3.4. Assessment of Hemolymph Immune, Antioxidant, and Biochemical Indicators

Table 6 shows immune and antioxidant parameters in hemolymph. Triglycerides (TGs) and the total protein (TP) level were significantly higher in the treated group compared to the control (0.00) group (*p* < 0.05). However, there were non-significant differences detected among treatments in total cholesterol (T-CHO), aspartate aminotransferase (AST), lysozyme (LZM), superoxide dismutase (SOD), complement component 3 (C3), and total antioxidant capacity (T-AOC) (*p* > 0.05). However, malondialdehyde (MDA) levels were highly significant in the control group compared to the treated group (*p* < 0.05).

### 3.5. Assessment of Hepatopancreas Antioxidant, Biochemical, and Immune Indicators

Table 7 shows antioxidant and immune indicators in the hepatopancreas. In the TB group, the levels of triglycerides (TGs) and total protein (TP) were significantly higher compared to the control group (*p* < 0.05). However, non-significant differences were detected among the treatments in total cholesterol (T-CHO), superoxide dismutase (SOD), and complement component 3 (C3) (*p* > 0.05).

### 3.6. Effect of TB on Textural Properties of Pacific White Shrimp Muscle

Table 8 presents the texture profile analysis (TPA) results, including the hardness, springiness, adhesiveness, chewiness, cohesiveness, resilience, and gumminess of the shrimp muscle. There were no significant differences detected among the groups in springiness, adhesiveness, cohesiveness, gumminess, resilience, or chewiness (*p* > 0.05). However, hardness was significantly high in the control group compared to the TB group (*p* < 0.05).

### 3.7. Intestinal Mucosal Morphology

**Figure 1:**
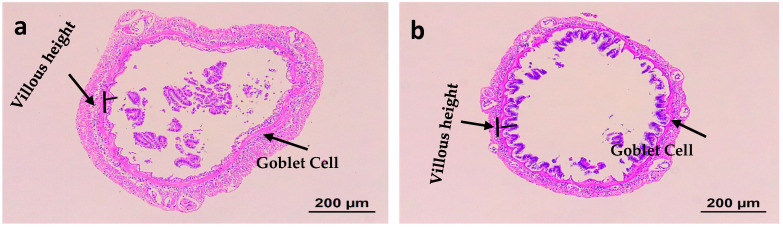
Effects of dietary tributyrin (TB) on structure of fore intestine in juvenile Pacific white shrimp (*Litopenaeus vannamei*) (10×). (**a**) Control group (0.00 g TB) showing anterior intestine villi. (**b**) TB group (2.24 g TB) showing anterior intestine villi.

Table 9 and Figure 1 present the intestinal mucosal morphology. Epithelial fold length was markedly higher in the TB-treated group (*p* < 0.05). However, non-significant differences were detected in the number of goblet cells per epithelial fold length between the groups.

### 3.8. The Histological Morphology of the Hepatopancreas

**Figure 2:**
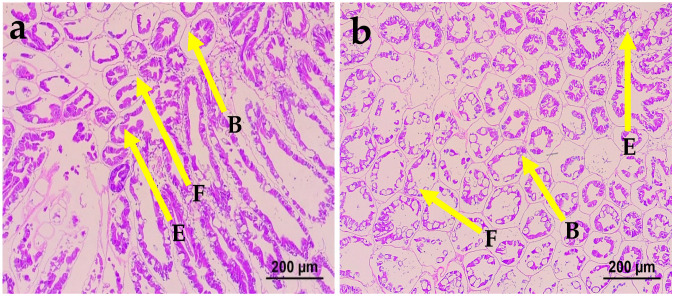
Representative histological photomicrographs of hepatopancreas from *L. vannamei* fed different diets (H&E staining, magnification ×200). (**a**) Shrimp fed control diet (control (0.00)), (**b**) shrimp fed treated diet (TB (2.24 g)). Letters B, E, and F in tubules indicate Embryonic cells (E-cells), Blister cells (B-cells), and Fibrillar cells (F-cells), respectively.

Figure 2 presents histological sections of the hepatopancreatic tubules from *Litopenaeus vannamei* fed with the diet. Embryonic cells (E-cells), Blister cells (B-cells), and Fibrillar cells (F-cells) were clearly identified in the hepatocytes of shrimp receiving the treated diet (b). In contrast, shrimp fed the control diet (a) exhibited hypertrophied B-cells in the hepatopancreas, whereas tributyrin (TB) (b) supplementation mitigated this hypertrophy. Shrimp fed the 2.24 g TB diet (b) displayed regular hepatocyte structures with regular cellular arrangement and had no abnormalities in the tubules.

## 4. Discussion

Tributyrin (TB) has been shown to improve the growth performance of aquaculture species receiving plant-based diets. Multiple studies have reported that the inclusion of TB in such diets significantly improves growth metrics in *Channa argus*, *Nibea albiflora*, *Acanthopagrus schlegelii*, *Scophthalmus maximus*, and hybrid grouper [29,30,31,32,33]. These growth-promoting effects are thought to be at least partly attributable to enhanced digestive efficiency and nutrient absorption through the modulation of intestinal function [34].

The present study investigated the effects of graded levels of tributyrin (TB) supplementation in a high-soybean-meal (SBM)-based diet on juvenile *Litopenaeus vannamei*. The results indicated that the inclusion of TB significantly enhanced the body weight (BW) of the shrimp. Although TB generally exerts beneficial effects, its effects on growth performance vary among species and depend on the dietary inclusion level, as reported in *Channa argus*, *Acanthopagrus schlegelii,* and *Megalobrama amblycephala* [29,31,35]. The present study also demonstrated that dietary supplementation with tributyrin (TB) significantly improved the SGR, FBW, and WGR in shrimp. The same results have been reported in *Scophthalmus maximus*, hybrid grouper, *Nibea albiflora*, *Cyprinus carpio*, and *Larimichthys crocea* [30,32,34,36,37,38]. The findings of Liu et al. (2017) [39] are consistent with our results, demonstrating that diet had no significant effect on the hepatosomatic index of shrimp.

In the present study, the reduced hepatosomatic index (HSI) observed in *Acanthopagrus schlegelii* indicates that the diets supplemented with tributyrin (TB) were nutritionally adequate and well balanced [40]. Although the highest growth performance was recorded in shrimp fed the TB-supplemented diet, this value was comparable to the optimal butyrate level reported for juvenile grass carp by Liu, Guo, Wu, Qu, Tan and Gong [39]. In the present study, the survival rate (SR), viscerosomatic index (VSI), and condition factor (CF) were not significantly affected in any experimental group. These results are consistent with those reported by [31,41] following the use of TB in *Acanthopagrus schlegelii*.

Digestibility is a key parameter for evaluating nutrient availability [42]. In the present study, apparent nutrient digestibility coefficients were higher in the tributyrin (TB)-supplemented group, in agreement with previous findings [43,44].

The nutritional value of shrimp is largely detected by their lipid and protein composition. In this study, shrimp fed with the control diet exhibited significantly lower protein and lipid concentrations in the dorsal muscle compared with individuals receiving TB-supplemented diets. Similarly, Ahmed and Sadek [45] reported that dietary sodium butyrate supplementation improved *Oreochromis niloticus* body composition. Nevertheless, the precise mechanisms by which tributyrin (TB) modulates body composition remain to be elucidated, underscoring the need for further investigation into its mode of action. In contrast, no significant alterations were detected in the proximate composition of either the dorsal muscle or whole body in the present study, which is consistent with the findings [46].

Physiological and biochemical indices are widely recognized as reliable indicators of shrimp health status and overall physiological function, as reported [47,48,49]. In the present study, dietary supplementation with tributyrin (TB) significantly influenced total protein (TP) concentrations in both hemolymph and the hepatopancreas. A similar effect has been reported in largemouth bass by Luo, Shi, Yang, Xu, Zhou, Gao, Wu and Wang [46]. In this study, the inclusion of TB in the diet significantly affected triglycerides (TGs) in hemolymph and the hepatopancreas, a finding previously reported in rabbit by Zhang et al. [50]. In the current study, dietary tributyrin (TB) supplementation non-significantly altered total cholesterol (T-CHO) levels in both the hemolymph and hepatopancreas, suggesting that TB does not adversely affect hepatopancreatic lipid metabolism and may contribute to maintaining hepatopancreatic health. This pattern aligns with earlier studies on rainbow trout [51] and Asian seabass [52].

Superoxide dismutase (SOD) is a key antioxidant enzyme that protects organisms from free radicals and species of reactive oxygen, thereby modifying oxidative stress and decreasing the risk of associated diseases, as reported by Shi and Zhou [53]. Malondialdehyde (MDA), a byproduct of lipid peroxidation and prostaglandin synthesis, serves as a marker of oxidative damage [54]. In this study, shrimp fed TB diets showed reduced MDA and increased SOD activity levels, while shrimp on the control diet exhibited the opposite trend. A comparable response has been reported in black sea bream [32], in broilers challenged with lipopolysaccharide following TB supplementation [55] and in mice with dextran sodium sulfate-induced colitis [56]. T-CHO was not affected by the diet, a result that was detected in broiler chickens in an earlier study [57]. Additionally, dietary treatment had no significant effect on AST or total cholesterol (T-CHO) levels, a result that was detected in Holstein calves in an earlier study [58].

These results propose that high-soybean-meal (SBM) diets can induce oxidative stress in Pacific white shrimp, which may be mitigated by tributyrin (TB) supplementation through an enhancement in hemolymph antioxidant capacity. Furthermore, Ma et al. [59] investigated the relationship between tributyrin and antioxidant capacity and reported that its protective effects against oxidative damage are partially mediated via the Nrf2 signaling pathway, which enhances the expression of antioxidant enzymes at the mRNA level.

Currently, most research focuses on investigating the effects of triglycerides on disease resistance, intestinal health, and the growth performance of different aquatic animals. However, their impact on the meat quality of Pacific white shrimp remains largely unexplored. Meat quality is a critical evaluation parameter for Pacific white shrimp, highly valued by consumers for its tender, flavorful, and minimally prickly flesh [60]. This quality encompasses multiple attributes, including nutritional composition, texture, flavor, and taste. In the present study, supplementation with 2.24 g of TB did not result in significant changes in the nutritional composition—including crude lipid, crude protein, ash, and moisture—compared with the control (CON) group. Amino acids are essential contributors to the taste and flavor profiles of foods. Our results indicated that the amino acid profiles of diets did not differ significantly between the 2.24 g group and the control (CON) group. Texture analysis revealed that supplementation with 2.24 g of TB did not affect any of the texture properties. Overall, supplementation with 2.24 g TB had a modest effect on the meat quality of *Litopenaeus vannamei*. The effects of tributyrin on meat quality observed in the present study were less pronounced than those reported previously, which may be related to differences in TB dosage and amino acid composition. More experiments are required to examine the effects of TB at varying concentrations.

The structural characteristics and morphology of intestinal villi are essential for efficient digestion and nutrient absorption [61]. In the present study, an increase in epithelial fold length was detected in the TB-supplemented groups. Alterations in intestinal histomorphology are commonly associated with gastrointestinal dysfunction. Previous studies in Atlantic salmon [62], Japanese flounder [63], and orange-spotted grouper [64,65] have demonstrated that diets containing high levels of soybean meal (SBM) or its derivatives can induce intestinal inflammation, primarily due to the occurrence of antinutritional factors (ANFs) in SBM [65]. Similar results were obtained by adding TB to the black sea bream, as reported by [31,41].

The hepatopancreas, which is functionally similar to the liver and pancreas in mammals, plays a key role in nutrient metabolism and overall health in crustaceans. In the present study, dietary replacement in the TB group’s diet led to the hypertrophy of B-cells. This observation is consistent with the findings reported by Burri et al. [66]. Previous studies have rarely reported the histomorphological effects of tributyrin supplementation on the hepatopancreas. In the present study, the normal hepatocyte structure and the regular arrangement of hepatopancreatic tubules observed in the tributyrin groups indicate that methionine supplementation restored hepatopancreatic integrity in *Litopenaeus vannamei* fed a low-fishmeal diet.

## 5. Conclusions

In the present study, we evaluated the effects of tributyrin on the growth performance, gut health, and flesh quality of Pacific white shrimp. The results indicated that supplementation with TB had modest but statistically significant effects on the growth performance and flesh quality of pacific white shrimp, including body weight, textural properties, amino acid profile, and proximate composition. However, TB significantly enhanced total protein, triglyceride, and epithelial fold length and improved gut health. The present study gives novel insights into application of tributyrin in aquaculture, supporting their potential use in aquaculture systems.

## Figures and Tables

**Table 1:** The formulation and proximate composition of experimental diets.

Ingredients	Diets	
	Control (0.00)	TB (2.24 g)
Fishmeal ^a^	100.0	100.0
Soybean meal ^a^	100.0	100.0
Fermented soybean meal ^a^	100.0	100.0
Soybean protein concentrate ^a^	170.0	170.0
Squid liver meal ^b^	30.0	30.0
Shrimp meal	50.0	50.0
Chicken meal	60.0	60.0
Fish oil	30.0	30.0
Beer yeast	40.0	40.0
Wheat flour	220.0	220.0
L-carnitine	2.0	2.0
Soybean lecithin	20.0	20.0
Carrageenan	3.7	3.7
Ascorbic phosphate ester	1.0	1.0
α-Starch	2.0	0.25
TB	0.00	2.24
*Yarrowia lipolytica*	2.0	2.0
Carboxymethyl cellulose	5.0	5.0
Mineral premix ^c^	5.0	5.0
Vitamin premix ^d^	3.0	3.0
Zeolite powder	21.0	21.0
L-Lysine	3.0	3.0
Ca(H_2_PO_4_)_2_	22.0	22.0
α-Cellulose	9.30	9.05
Butyrin	1.0	1.0
Total	1000.0	1000.0
Proximate composition		
Moisture (%)	10.64	12.59
Crude protein (%)	47.73	47.99
Crude lipid (%)	7.57	7.89
Crude ash (%)	13.58	13.37
Gross energy (kJ g^−1^)	17.94	17.91

^a^ The fishmeal used in this study was obtained from Jin Jia Co., Ltd., located in Hangzhou, China. ^b^ The soybean meal used in this study was obtained from Sangon Biotech Co., Ltd., based in Shanghai, China. ^c^ Supplied by NHU Amino Acid Co., Ltd., Binhai district, Weifang, China. ^d^ The composition of the vitamin premix (mg kg^−1^) is as follows: 80 mg of retinyl acetate, 40 mg of α-tocopherol, menadione 0.1 mg, cholecalciferol 15 mg, niacin 165 mg, 22 mg of riboflavin, 40 mg of HCl, 45 mg of D-Ca pantothenate, 102 mg of pyridoxine, 40 mg of thiamine mononitrate, 0.9 mg of vitamin B12, 10 mg of folic acid, 150 mg of ascorbic acid, 450 mg of inositol, 5 mg of thiamine, 15 mg of sodium menadione bisulfate, 50 mg of p-aminobenzoic acid, and 320 mg of choline chloride.

**Table 2:** The amino acid composition of experimental diets.

Amino Acids	Diet No.	
	Control (0.00)	TB (2.24 g)
EAA		
Threonine	1.261	1.687
Valine	1.733	3.058
Methionine	0.373	0.593
Isoleucine	1.426	2.474
Leucine	2.771	1.699
Phenylalanine	1.614	2.785
Lysine	2.641	3.560
Histidine	0.844	1.460
Arginine	2.237	3.894
NEAA		
Alanine	1.845	3.272
Aspartate	1.649	3.886
Cysteine	0.096	0.178
Glutamic acid	0.052	0.071
Glycine	2.058	3.544
Proline	1.931	1.942
Serine	0.536	1.110
Tyrosine	0.763	1.284

Values for the proximate analysis of the test diets are the means of triplicate analyses. The amino acid values reported in this table are based on experimental measurements. Abbreviations: TB, tributyrin. (i) Essential amino acids (EAAs): Histidine, valine, methionine, phenylalanine, arginine, lysine, threonine, leucine, and isoleucine. (ii) Non-essential amino acids (NEAAs): Glycine, aspartate, tyrosine, glutamic acid, alanine, proline, serine, and cysteine.

**Table 3:** Effect of TB on growth and feed utilization in juvenile Pacific white shrimp (*n* = 3).

Parameters	Diets	
	Control (0.00)	TB (2.24 g)
IBW	1.66 ± 0.19	1.43 ± 0.11
FBW	7.12 ± 0.05 ^b^	7.60 ± 0.13 ^a^
WG (%)	329.52 ± 9.02 ^b^	429.58 ± 8.05 ^a^
SGR (% d^−1^)	2.58 ± 0.12 ^b^	2.98 ± 0.04 ^a^
MFI (g shrimp^−1^ day^−1^)	0.34 ± 0.01	0.33 ± 0.01
FCR	2.81 ± 0.15	2.50 ± 0.37
CF (%)	1.16 ± 0.05	1.25 ± 0.22
HSI (%)	3.88 ± 0.06 ^a^	3.65 ± 0.05 ^b^
VSI (%)	5.14 ± 0.13 ^a^	4.73 ± 0.14 ^b^
SR (%)	97.50 ± 2.50	97.33 ± 0.94

Values are presented as the mean ± SD of three replicate aquaria (*n* = 3). Values with different superscript letters within the same row indicate significant differences (*p* < 0.05).

**Table 4:** The effects of dietary tributyrin (TB) levels on the apparent digestibility coefficients (%) of nutrients in the feces of juvenile Pacific white shrimp over 8 weeks.

Parameters	Diets	
	Control (0.00)	TB (2.24 g)
Crude protein	86.21 ± 0.59	86.42 ± 0.43
Crude lipid	87.52 ± 0.94	87.63 ± 0.54
Ash	38.23 ± 1.11 ^a^	33.72 ± 0.88 ^b^
Gross energy (%)	83.90 ± 0.86	83.87 ± 0.91

Values are presented as the mean ± SD of three replicate aquaria (*n* = 3). Values with different superscript letters within the same row indicate significant differences (*p* < 0.05).

**Table 5:** Proximate composition (on wet weight basis) of whole body and muscle in juvenile Pacific white shrimp (*Litopenaeus vannamei*) fed experimental diets.

Parameters	Diets	
	Control (0.00)	TB (2.24 g)
Whole body		
Crude protein (%)	17.32 ± 0.51	17.23 ± 0.19
Crude lipid (%)	1.60 ± 1.25	1.21 ± 0.18
Moisture (%)	78.58 ± 2.24	76.79 ± 2.59
Ash (%)	4.61 ± 0.12 ^a^	4.45 ± 0.17 ^b^
Phosphorus (%)	1.25 ± 0.19	1.25 ± 0.03
Dorsal muscle		
Crude protein (%)	20.79 ± 0.19	20.81 ± 0.47
Crude lipid (%)	0.39 ± 0.36 ^b^	1.29 ± 0.33 ^a^
Moisture (%)	76.24 ± 0.51	75.24 ± 0.87
Ash (%)	1.72 ± 0.08	1.72 ± 0.09
Phosphorus (%)	2.76 ± 0.24	2.63 ± 0.61

Values are presented as the mean ± SD of three replicate aquaria (*n* = 3). Values with different superscript letters within the same row indicate significant differences (*p* < 0.05).

**Table 6:** Hemolymph, biochemical, immune, and antioxidant indictors of juvenile Pacific white shrimp fed experimental diets for eight weeks (*n* = 3).

Parameters	Diets	
	Control (0.0)	TB (2 g)
TP (g L^−1^)	1.77 ± 0.15 ^b^	2.06 ± 0.05 ^a^
TG (mmol L^−1^)	2.06 ± 0.02 ^b^	2.33 ± 0.12 ^a^
T-CHO (mmol L^−1^)	1.69 ± 0.04	1.69 ± 0.06
AST (U L^−1^)	10.88 ± 0.33	10.74 ± 0.13
LZM (U mL^−1^)	60.47 ± 0.41	60.27 ± 0.24
T-AOC (U mL^−1^)	1.41 ± 0.12	1.29 ± 0.40
SOD (U mL^−1^)	10.55 ± 0.47	11.21 ± 0.77
MDA (nmol mL^−1^)	3.74 ± 0.58 ^a^	2.55 ± 0.19 ^b^
C3 (μg mL^−1^)	63.49 ± 3.63	65.78 ± 0.34

Values are presented as the mean ± SD of three replicate aquaria (*n* = 3). Values with different superscript letters within the same row indicate significant differences (*p* < 0.05). Abbreviations: TP, total protein; MDA, malondialdehyde; T-AOC, total antioxidant capacity; TG, triglyceride; LZM, lysozyme; AST, aspartate aminotransferase; T-CHO, total cholesterol; C3, complement protein 3; SOD, superoxide dismutase.

**Table 7:** Antioxidant and biochemical indicators in hepatopancreas of juvenile Pacific white shrimp (*Litopenaeus vannamei*) fed with experimental diets for eight weeks (*n* = 3).

Parameters	Diets	
	Control (0.00)	TB (2.24 g)
TP (mg g^−1^ tissue)	1.21 ± 0.04 ^b^	1.76 ± 0.17 ^a^
TG (mg g^−1^ tissue)	1.09 ± 0.01 ^b^	1.28 ± 0.11 ^a^
T-CHO (mg g^−1^ tissue)	1.07 ± 0.01	1.07 ± 0.07
SOD (U g^−1^ tissue)	6.53 ± 0.43	7.15 ± 0.21
C3 (μg g^−1^ tissue)	37.12 ± 4.33	37.10 ± 3.75

Values are presented as the mean ± SD of three replicate aquaria (*n* = 3). Values with different superscript letters within the same row indicate significant differences (*p* < 0.05). Abbreviations: SOD, total antioxidant capacity; T-CHO, total cholesterol; TG, triglyceride; TP, total protein; C3, complement protein 3.

**Table 8:** Effect of TB on textural properties of Pacific white shrimp muscle (*n* = 3).

Parameters	Diets	
	Control (0.00)	TB (2.24 g)
Hardness (g)	123.38 ± 1.05 ^a^	114.26 ± 2.99 ^b^
Adhesiveness (g)	−49.44 ± 32.52	−32.83 ± 11.94
Springiness	0.83 ± 0.09	0.85 ± 0.13
Cohesiveness	0.28 ± 0.03	0.35 ± 0.03
Gumminess (g)	36.52 ±7.15	46.42 ± 18.37
Chewiness (g)	38.91 ± 0.47	34.16 ± 2.13
Resilience	0.03 ± 0.01	0.06 ± 0.02

Values are presented as the mean ± SD of three replicate aquaria (*n* = 3). Values with different superscript letters within the same row indicate significant differences (*p* < 0.05).

**Table 9:** Effect of dietary tributyrin (TB) levels on structure of intestinal mucosa in juvenile Pacific white shrimp.

Parameters	Diets	
	Control (0.00)	TB (2.24 g)
Epithelial fold length (μm)	60.67 ± 0.58 ^b^	63.24 ± 0.55 ^a^
Number of goblet cells/Epithelial fold length	0.83 ± 0.01	0.85 ± 0.02

Values are presented as the mean ± SD of three replicate aquaria (*n* = 3). Values with different superscript letters within the same row indicate significant differences (*p* < 0.05).

## Data Availability

All original findings from this study are presented in the article, and any further questions can be addressed to the corresponding authors.
